# Absolute quantitative real-time polymerase chain reaction for the measurement of human papillomavirus E7 mRNA in cervical cytobrush specimens

**DOI:** 10.1186/1750-9378-2-8

**Published:** 2007-04-02

**Authors:** Michael E Scheurer, Laura M Dillon, Zhuo Chen, Michele Follen, Karen Adler-Storthz

**Affiliations:** 1 The University of Texas M. D. Anderson Cancer Center, Department of Epidemiology, Unit 1340, PO Box 301439, Houston, TX 77230-1439, USA; 2 The University of Texas Dental Branch, Department of Diagnostic Sciences, 6516 MD Anderson Blvd, DBB 4.133, Houston, TX 77030, USA; 3 Emory University Winship Cancer Institute, Department of Hematology and Oncology, 1365 Clifton Road, Building C, Suite C3086, Atlanta, GA 30322, USA; 4 The University of Texas M. D. Anderson Cancer Center, Department of Gynecologic Oncology and Biomedical Engineering Center, Unit 193, 1515 Holcombe Blvd., Houston, TX 77030, USA

## Abstract

**Background:**

Few reports of the utilization of an accurate, cost-effective means for measuring HPV oncogene transcripts have been published. Several papers have reported the use of relative quantitation or more expensive Taqman methods. Here, we report a method of absolute quantitative real-time PCR utilizing SYBR-green fluorescence for the measurement of HPV E7 expression in cervical cytobrush specimens.

**Results:**

The construction of a standard curve based on the serial dilution of an E7-containing plasmid was the key for being able to accurately compare measurements between cervical samples. The assay was highly reproducible with an overall coefficient of variation of 10.4%.

**Conclusion:**

The use of highly reproducible and accurate SYBR-based real-time polymerase chain reaction (PCR) assays instead of performing Taqman-type assays allows low-cost, high-throughput analysis of viral mRNA expression. The development of such assays will help in refining the current screening programs for HPV-related carcinomas.

## Background

Infection with certain types of human papillomavirus (HPV), while a necessary cause of cervical cancer, does not guarantee an elevated risk of malignancy. An estimated 80% of all women will become infected with HPV at some point in their lives [1]; however, few will develop dysplasia of the cervix. Approximately 11% of those that develop mild dysplasia or low-grade squamous intraepithelial lesions (LSIL) will progress to severe dysplasia or high-grade squamous intraepithelial lesions (HSIL), and an equal fraction will further progress to invasive carcinoma [2]. The challenge is determining which women are likely to progress versus those who will spontaneously regress. While screening with the Papanicolaou (Pap) test has been effective in lowering cervical cancer rates in countries with effective screening systems, it is still not sensitive and specific enough to accurately predict the behavior of pre-cancerous lesions.

Laboratory studies have revealed that uncontrolled expression of the HPV E6 and E7 oncogenes is responsible for the ability of HPV to transform cells. While, the functions of the E6 and E7 proteins together can immortalize human foreskin keratinocytes [3], those of E6 alone cannot [4]. In addition, the E7 oncogene is more highly conserved upon viral integration than is E6 [5,6]. The E7 oncogene exerts its transforming function by interrupting cell differentiation and inducing DNA synthesis [7]. It accomplishes this by interacting with the cellular tumor suppressor gene product pRB [8,9]. Additionally, E7 can induce abnormal centrosome duplication [10,11] and chromatin condensation [12] possibly leading to chromosome instability.

E7 mRNA has been shown to be present in basal layers in organotypic raft cultures [13], and higher levels have been found in cervical tumors [14,15]. Few studies [16-19] have looked for the presence of viral transcripts in precancerous clinical specimens. These studies have shown an increase in prevalence of HPV E6 and/or E7 mRNA transcripts, and Wang-Johanning et al. [19], in particular, reported that copy number of both RNA and DNA increased with increasing SIL grade.

We were interested in determining if any quantitative differences existed in the expression of E7 mRNA in cells from women with normal epithelium and precancerous lesions. In order to accomplish this goal, we needed to develop a polymerase chain reaction (PCR) method that would allow us to make direct comparisons between patients in terms of amount of mRNA expressed in cervical specimens. Here, we describe the development of an absolute quantitative real-time PCR assay to quantitate mRNA of the E7 oncogene from HPV 16 and HPV 18 in cervical cytobrush samples.

## Results

### Absolute quantitation of mRNA

Eighteen cytobrush specimens were analyzed by real-time PCR for the presence of either HPV16 or HPV18 E7 mRNA (Table 1). Thirteen samples (87%) showed the presence of E7 mRNA. The others exhibited no evidence of HPV E7 mRNA in either replicate. Samples 5, 8, and 9 were included as specificity controls. Samples 5 and 8 were HPV16 and HPV18 negative but consensus positive by DNA PCR; sample 9 was negative by all three probes (HPV16, HPV18, and consensus). All of these samples produced negative results in the quantitative real-time PCR; showing that the primers used were specific for HPV16 or HPV18. As a comparison, the copy numbers of the control samples (SiHa and HeLa) were calculated in each reaction. These quantities were then averaged over the runs to show a mean transcript copy number of 10.9 per 20 ng cDNA for HPV16 in the SiHa samples, and a mean transcript copy number of 82.6 per 20 ng cDNA for HPV18 in the HeLa samples. These values are comparable to previously reported values for SiHa and HeLa. [20,21]

**Table 1:** Results of Repeated Quantitative Real-time PCR on Cytobrush Specimens

ID	HPV type assayed	mRNA Log Starting Quantity***		mRNA Copy Number****	
		
		Repeat 1	Repeat 2	Repeat 1	Repeat 2
1	16	1.3845	1.409	3.99	4.09
2	16	0	0	0	0
3	16	0.7095	1.026	2.03	2.79
4	16	0	0	0	0
5*	16	0	0	0	0
6	16	1.5385	1.274	4.66	3.58
7	16	1.4075	1.4615	4.09	4.31
8*	16	0	0	0	0
9**	16	0	0	0	0
10	16	1.5605	1.6785	4.76	5.36
11	16	0.737	0.8855	2.09	2.42
12	16	0	0.253	0	1.29
13	16	0.6945	0.656	2.00	1.93
14	16	0.662	0.5	1.94	1.65
15	16	1.35	1.4165	3.86	4.12
16	16	0.468	0.458	1.60	1.58
17	18	0.6625	1.0695	1.94	2.91
18	18	4.2175	4.1265	67.86	61.96

Note: Coefficient of Variation (CV) = 10.4%

*HPV16 and HPV18 negative, HPV consensus positive; used as specificity control

**HPV16, HPV18, and HPV consensus negative; used as specificity control

***Log SQ = ln(copy number)

****per 20 ng cDNA

### Reproducibility of real time-PCR

Reproducibility of the PCR assay was determined by repeating the assay on two separate days one week apart. Since we started the PCR step with the same amount of total cDNA from each specimen, we were more concerned with the reproducibility of the PCR step rather than the Reverse Transcription step. As previously stated, the Reverse Transcription step was performed separately from the PCR step to ensure that the quantity of each sample assayed was equivalent. A one-factor random-effects analysis of variance was performed to estimate the coefficient of variation. A coefficient of variation of <20% is considered adequate for the reproducibility of the assay [22]. All statistical analyses were performed using Intercooled STATA 8.2 (Stata Corp., College Station, TX). The coefficient of variation calculated for this assay was 10.4%.

**Figure 1:**
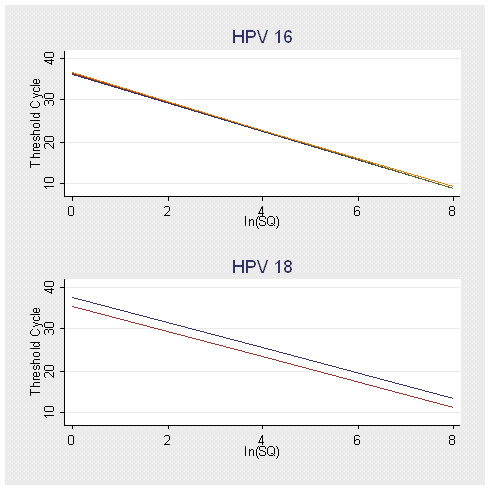
Comparison of Standard Curves from Repeated Assays by HPV Type. This figure is a graphical comparison of the standard curves from each of the HPV 16 and HPV 18 assays. There were four assays performed for HPV 16 and two for HPV 18. The type-specific curves were not statistically different from one another, and those for HPV 16 are graphically identical. (Note: ln(SQ) = natural log of starting quantity.)

In addition, the standard curves from each repeated assay by HPV type were compared utilizing a linear regression model to fit the threshold cycle (C_T_) values to variables representing the natural log of the copy number, the repeat run number, and an interaction term of the run number with the natural log of the copy number. This model allows tests of parallelism and coincidence for the standard curves from each run. If the p-value from the test for parallelism is not significant at the α = 0.05 level, then the regression lines are parallel. Similarly, if the p-value from the test for coincidence is not significant at the α = 0.05 level, then the regression lines are coincident. The regression lines for each HPV type were compared separately, and there were no significant differences between them (see Table 2). This can also be seen graphically in Figure 1. As a result, we are confident that for each HPV type, the assay itself is reproducible with great accuracy.

**Table 2:** Comparison of Standard Curves from Real-time PCR Repeat Runs

Run	r^2^	Slope	Intercept	Efficiency	P_parallel_	P_coincident_
HPV16-1	0.994	-3.386	35.878	97.4%	0.9640	0.8790
HPV16-2	0.997	-3.434	36.290	95.5%		
HPV16-3	0.996	-3.450	36.423	94.9%		
HPV16-4	0.997	-3.442	36.536	95.2%		
						
HPV18-1	0.966	-2.999	37.714	115.5%	0.8753	0.1479
HPV18-2	0.990	-3.054	35.629	112.5%		

Note:

P_parallel _tests that the curves are parallel to one another (i.e., they have the same slope)

P_coincident _tests that the curves are coincident (i.e., they have the same slope and y-intercept)

### Quality of cDNA

To assess the quality of the cDNA for RT-PCR, all negative samples (n = 5) and a subset of the positive samples (n = 5) were analyzed by real-time PCR using SYBR Green I for the presence of the G3PDH gene. All samples were positive for G3PDH; therefore, we are reasonably certain that the cDNA was of sufficient quality to detect HPV mRNA, if present.

## Discussion

To our knowledge this is only the second report of an absolute quantitative real-time PCR to measure levels of HPV mRNA and the first to do so using less expensive SYBR green methods. An earlier report of the use of quantitative PCR by Wang-Johanning et al. utilized a one-step, Taq-based PCR assay [19]. Our assay differs from theirs in two ways. First, we used a two-step assay separating the RT step from the PCR, in this way we were able to measure the amount of RNA transcript in exactly 20 ng of total cDNA. Second, our assay utilized the SYBR Green I intercalating dye, which allowed us to lower the overall cost of the assay.

While generally considered more specific, the high cost of TaqMan-based PCR reactions could be prohibitive for large-scale molecular epidemiological studies and structured screening programs. Compared to relative PCR methods, the use of absolute real-time PCR allows a direct comparison of the exact copy number of E7 mRNA in each sample. Other results reported in the literature utilized relative copy numbers in regards to a reference gene, typically the G3PDH gene. Relative quantitative PCR reports the number of copies of the gene of interest in relation to the number of copies of the reference gene present in the reaction. This helps in identifying the viability of the sample to produce results; however, the results should be normalized to an equivalent level of reference gene if comparisons between samples are to be made. The absolute quantitative PCR reported here allows a direct comparison of the copy numbers of the gene of interest without the need for a reference gene through the construction of the standard curve in each assay. We have shown here that the standard curve for the reaction is highly reproducible and allows accurate comparisons of HPV E7 mRNA between cytobrush samples.

It is feasible that the detection of high-risk HPV transcripts could serve as a marker for risk of the development of cervical cancer. It has been shown that E7 promotes the formation of benign lesions while E6 works to complete the malignant transformation [4]. If the factors that contribute to the ability of E7 to advance benign lesions were identified, there might be a way to either identify women at risk for more intensive screening versus those in whom the screening process could be minimized. The only other main viral factor that has been examined as a risk factor for cervical cancer progression is viral load; however, evidence is still unclear. Most studies associate a high viral load with high-grade cervical lesions [23-28]; however, more recent studies suggest that it is a predictor of low-grade lesions [28-30] and ASCUS [28] as well.

The addition of molecular testing, such as this, to Pap testing could increase the effectiveness of cervical cancer screening programs by increasing the limited sensitivity (58%) and specificity (69%) [31] of the Pap test and the resulting low predictive values of carcinoma. Some progress has been made with the addition of liquid-based cytology or HPV DNA testing to conventional Pap testing [32]. However, simply detecting the presence of viral DNA is not likely to add as much information as the testing for viral persistence evidenced by integration and expression of viral oncogenes. The HPV E7 oncogene is the most conserved viral gene after integration. Therefore, testing for E7 mRNA, over E6 or E5, could better prove integration has occurred, the virus is being maintained in the cell, and its gene products are affecting the cellular replication machinery. It is this persistent infection that can lead to the progression of SIL to high-grade lesions and squamous cell carcinoma.

There are several advantages to using real-time PCR for this purpose. First, typically with cervical sampling very small amounts of cells or tissue are retrieved. Real-time PCR can quantitate gene expression from very small amounts of starting material. Also the ease of the procedure lends itself to use in large-scale epidemiologic studies where many hundreds of subjects can be analyzed. Older studies utilizing fluorescent *in situ *hybridization and radio-labeled blotting techniques were too labor-intensive for these large studies [33] and lacked the sensitivity needed to detect the transcripts in low-grade and normal tissues [34].

Methods to reuse collected material or combine tests would greatly enhance the yield from cervical samples for future studies. An example of this is the use of liquid-based cytology to perform Pap smears, after which the cells are available for HPV typing [35]. In addition, continuing efforts to improve the reliability of multiplex real-time PCR reactions will allow the testing of multiple HPV types in one assay. This would further decrease the amounts of cellular material needed for these studies. This is extremely important in the cervix where few viable cells are collected during sampling.

## Conclusion

In summary, we present here the methods used to develop an absolute quantitative real-time PCR for the measurement of expression of viral oncogenes. We showed previously that this method could be utilized in a high-throughput manner to assess HPV E7 expression in clinical samples[36]. The ability to directly measure these oncogenes could facilitate the discovery of markers that predict cervical progression. The main goal of this type of research is to hone the screening capabilities for HPV-related carcinoma. Several suggestions have been made as additional markers to include in cervical cancer screening (HPV typing, HPV viral load, etc.); however, much research is still needed to meet the goal of a highly sensitive and specific screening program.

## Methods

### Cervical samples

Cervical specimens were collected as part of a larger study to evaluate emerging optical technologies for cervical neoplasia. The study population consisted of women with abnormal Pap test results attending the colposcopy clinics at The University of Texas M. D. Anderson Cancer Center, Lyndon B. Johnson General Hospital, and Memorial Hermann Hospital in Houston, Texas, USA, and the British Columbia Cancer Agency in Vancouver, British Columbia, Canada, between October 2000 and July 2003. IRB approval was received from each institution involved in the study, and participants gave written informed consent prior to being enrolled in the study.

Clinical specimens were collected for various laboratory procedures, including: histopathologic confirmation of disease, quantitative cyto- and histopathology, HPV typing, and HPV DNA and mRNA analyses. Study physicians collected specimens for HPV DNA and RNA analyses using an endocervical cytobrush. Specimens for DNA extraction were placed in 250 μl PBS with 0.02% sodium azide immediately following cytologic sampling and stored at -80°C until extracted, approximately one month after collection. Specimens for RNA extraction were placed in 250 μl of lysis buffer (Ambion, Austin, TX) immediately following cytologic sampling, and stored at -80°C until extracted; length of storage varied from 5 months to 3 years after collection. To assess the quality of the mRNA, the concentration of all mRNA samples was measured by spectrophotometry, and all cDNA samples were assayed for G3PDH by real-time PCR. All samples were measurable by both methods.

### Cell lines

The cervical cancer cell lines HeLa and SiHa were purchased from American Type Culture Collection (Manassas, VA) for use as positive controls in the reactions. HeLa cells express HPV 18 mRNA, and SiHa cells express HPV 16 mRNA. Both cell lines were grown in Eagle's Minimal Essential Medium (EMEM) with 10% fetal bovine serum and penicillin-streptomycin. The cells were incubated at 37°C in a 5% CO_2 _atmosphere and harvested directly in lysis buffer (Ambion, Austin, TX). Total mRNA was isolated from the harvested cells using the same kit used for the patient samples (RNAqueous, Ambion, Austin, TX), and cDNA was similarly generated (RETROScript, Ambion, Austin, TX) (see section *RNA extraction and reverse transcription*).

### DNA isolation and detection

Viral DNA was extracted from cervical cytobrush specimens using a commercially available kit (QIAamp DNA Mini Kit, Qiagen, Valencia, CA, USA) according to manufacturer's buccal swab spin protocol. To maximize DNA yield from the cervical samples, all 250 μl of lysate was used for DNA extraction. Briefly, 20 μl of Proteinase K and 400 μl of Buffer AL was added to the tube containing the specimen. The reaction was incubated at 56°C for 10 minutes, and 400 μl of ethanol was added to the tube and vortexed to mix. Immediately prior to elution of the DNA, the cytobrushes were removed from the tubes by using forceps, which were flamed between each use to prevent contamination. The cytobrush was left in the tube up to this point to harvest as much of the DNA as possible from the sample. This mixture was applied in two steps to the QIAamp Spin Column and centrifuged at 8,000 rpm for 1 min after each application. The spin column was washed with 500 μl of Buffer AW1 and AW2, centrifuging for 1 min at 8,000 rpm and 3 min at 14,000 rpm, respectively. Any fluid was removed from the collection tube and the column was centrifuged for 1 min at 14,000 rpm to remove any residual fluid and dry the filter. The filter was placed in a clean microcentrifuge tube, and 110 μl of Buffer AE was added to the filter. This was incubated at room temperature for 5 min, and then eluted by centrifuging for 1 min at 8,000 rpm. Extracted DNA was stored at -80°C for no more than 4 weeks before PCR was performed.

Following the methods of Manos et al. [37], we then analyzed the samples for HPV DNA using MY9 and MY11 consensus HPV primers that amplify a 450 base pair region of the L1 open reading frame of at least 28 different HPV types. PCR products were resolved by agarose gel electrophoresis, transferred to nylon membranes (Bio-Rad Laboratories, Hercules, CA), and hybridized to a ^32^P-labeled HPV consensus probe. Consensus probe-positive samples were then hybridized to ^32^P-labeled specific HPV 16 and HPV 18 probes on separate nylon membranes. Sample positivity was assessed by autoradiography following hybridization. DNA extracted from HPV 18-positive HeLa cells, HPV 16-positive CaSki cells, and a negative control without DNA were used as controls in all the PCRs and subsequent hybridizations.

### RNA extraction and reverse transcription

RNA was extracted as total mRNA from cervical cytobrush specimens using a commercially available kit (RNAqueous, Ambion, Austin, TX). To increase the mRNA yield, all 250 μl of the lysate was used for mRNA extraction. Briefly, 125 μl of 100% ethanol was added to the sample tube, which was then briefly vortexed and centrifuged. The cytobrushes were then removed from the tubes after ethanol treatment by using forceps, which were flamed between each use to prevent contamination. The lysate mixture was loaded in 150-μl aliquots onto a Micro Filter Cartridge tube and centrifuged to pass the solution over the filter. This was repeated until all of the lysate was passed through the filter. Wash solutions (1 and 2/3) were added to the filter, and the tube was centrifuged between each wash. Any fluid was removed from the collection tube, and the filter was centrifuged for 1 min to remove any residual fluid and dry the filter. The filter was placed in a clean Micro Elution tube, and the RNA was eluted into a total of 20 μl of elution solution heated to 75°C in 2 steps, centrifuging at each step. Treatment with DNase I was done according to kit instructions to remove any trace amounts of contaminating genomic DNA. Following inactivation of the DNase, the tube was centrifuged for 2 min, and the pellet was left in the tube, which was then stored at -80°C.

For the expressed purposes of analyzing the same amount of cDNA in every specimen, reverse transcription was performed as a separate step from PCR. The mRNA was then reverse transcribed into cDNA (RETROscript, Ambion, Austin, TX) on the same day as the extraction. Briefly, 3 μl of random decamers (provided in kit) was added to 15 μl of the RNA elution mixture. A final reaction volume of 30 μl was achieved with the addition of 3 μl 10× RT Buffer, 6 μl dNTP mix, 1.5 μl RNase Inhibitor, and 1.5 μl reverse transcriptase (all provided in kit). The reaction was vortexed briefly and incubated at 50°C for 1 hr. The tubes were then heated at 92°C for 10 minutes to inactivate the reverse transcriptase. To obtain the highest yield of cDNA, the incubation step was optimized at 50°C (from the standard 44°C) per the recommendation of the manufacturer. The cDNA samples were then purified using NucAway Spin Columns (Ambion, Austin, TX) to remove unused dNTPs and any other DNA or RNA material <25 bases in length. The concentration of the cDNA was determined in a spectrophotometer at 260 nm on the same day it was generated, and the cDNA was stored at -80°C for no more than 1 month prior to analysis by real-time PCR.

### Absolute real-time PCR

**Figure 2:**
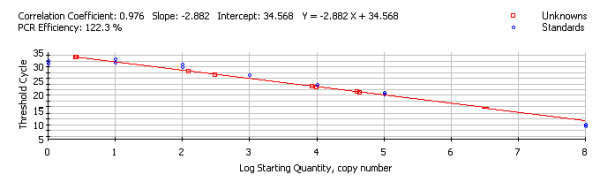
Standard Curve from HPV 16 E7 Assay. This figure represents the standard curve of the HPV 16 PCR. Note, the standard dilutions appear as blue circles on the graph, and the unknown samples appear as red squares. The calculated regression line of the curve is given above the graph. In this case, Y = -2.882X + 34.568. Setting Y equal to the C_T _for the unknown, solving for X will give the starting quantity for the unknown sample.

To perform an absolute quantitative real-time PCR, plasmids constructed with specific binding sites for HPV 16 and HPV 18 E7 primers were used to derive the standard curves (Figure 2) for each set of patient samples that were assayed [38]. Reactions were carried out for HPV 16 and HPV 18 separately, and each experiment had its own standard curve. The following primers were used for construction of the HPV 16 E7 and HPV 18 E7 internal standard with human glyceraldehyde-3-phosphate dehydrogenase (G3PDH) cDNA as a template. These primers contained 24 bases of HPV 16 or 18 E7 sequence (shown as capital letters) and 20 bases of G3PDH sequence (shown as lower-case letters).

HPV 16:5'-ATGACAGCTCAGAGGAGGAGGATGacggatttggtcgtattggg-3'

5'-CAGATGGGGCACACAATTCCTAGTtgattttggagggatctcgc-3'

HPV 18:5'-CACGAGCAATTAAGCGACTCAGAGacggatttggtcgtattggg-3'

5'-ATGCAGACCACGGACACACAAAGGtgatttggagggatctcgc-3'

PCR was performed using these primers and G3PDH cDNA as a template. The resulting PCR products contained 24 base pairs of HPV 16 E7 or HPV 18 E7 at each end of a 278 G3PDH base pair product. These PCR products were then reamplified using the same HPV sequences used in the first round of PCR, but without the G3PDH sequences. The resulting products, one for HPV 16 E7 and one for HPV 18 E7, were ligated into a pCRII plasmid (Invitrogen, San Diego, CA). Sequence analysis of the two recombinant plasmids confirmed that the HPV sequences were correctly attached at the end of the G3PDH fragment. The utility of the plasmid is two fold: to perform absolute quantitative PCR from a standard curve based on copy number, and to have the ability to multiplex HPV and G3PDH in the same reaction, although this was not done for the current study.

A set of serial dilutions of the HPV E7-containing plasmids (1 × 10^8^, 1 × 10^5^, 1 × 10^4^, 1 × 10^3^, 1 × 10^2^, 1 × 10^1^, 1 × 10^0 ^copies/μl) was used as PCR templates for the standard curve. Each standard dilution was tested in triplicate, and each patient sample was tested in duplicate (due to the minimal yield of the mRNA extraction process). Positive (SiHa for HPV 16 and HeLa for HPV 18) and negative controls were included in each experiment to ensure reproducible results.

The PCR technique used in this study was an absolute quantitative real-time PCR assay using the SYBR Green I (Molecular Probes, Eugene, OR) fluorescent intercalation dye. Each amplification experiment was performed in a 96-well PCR plate covered with optical tape in the iCycler iQ real-time PCR instrument (Bio-Rad Laboratories, Hercules, CA). A final volume of 25 μl was used containing 20 ng of cDNA template, 12.5 μl of iQ SYBR Green Supermix (Bio-Rad Laboratories, Hercules, CA), 1.25 μl containing 0.2 micromoles of either HPV 16 or HPV 18 forward and reverse primers, and water. Primers used for HPV 16 E7 PCR were 5'-atgacagctcagaggaggaggatg-3' and 5'-cagatggggcacacaattcctagt-3'. They were designed to amplify a 196 bp fragment of HPV 16 E7 cDNA (nt 647-nt 842). Primers for HPV 18 E7 PCR were 5'-atgcagaccacggacacacaaagg-3' and 5'-cacgagcaattaagcgactcagag-3' which amplified a 226 bp fragment of HPV 18 E7 cDNA (nt 671-nt 896). A BLAST search of the primers used for HPV 16 E7 showed alignment with all major variants of HPV 16 (AA, Af1, Af2, As, and E). There is less information in the NCBI database on HPV 18 since it is much less frequent than HPV 16. A BLAST search of the primers used for HPV 18 E7 showed alignment with HPV 18 sequences; however, the variant specificity was not available. Although variant-specific sequences for HPV 18 were not available for comparison, it has been shown that the E6 and E7 genes of the high-risk HPVs are highly conserved among viral types and would therefore, be highly conserved among the variants within the types. [39] We, therefore, do not expect that variants of HPV 18 would cause problems in quantifying the E7 gene in our samples. Reaction contamination by exogenous HPV DNA or RNA was ruled out by the inclusion of a reaction tube containing all reagents but no sample cDNA. The reaction was subjected to denaturation at 95°C for 2 minutes followed by 40 cycles of denaturation at 95°C for 45 seconds, annealing at 62°C for 45 seconds, and elongation at 72°C for 45 seconds. Fluorescent data were specified for collection at the end of the elongation step in each cycle. SYBR Green I binds and intercalates into double-stranded DNA during the extension step of the amplification cycle.

An equal quantity of cDNA (20 ng) from each sample was analyzed by real-time PCR for quantitation of HPV 16 and HPV 18 E7 oncogene expression (cDNA levels reflect mRNA levels). Using the same amount of total cDNA (20 ng) from each sample allows for accurate comparison of the target mRNA between the samples independent of amount of sample collected and independent of the efficiency of the RT step. This was the purpose of carrying out the two-step real-time PCR. All study participants were identified as positive for HPV 16 and/or HPV 18 DNA by PCR. Three additional samples that were HPV16 and HPV18 DNA negative, but two were positive by consensus hybridization and one negative by consensus hybridization. These samples were included to test the viral type specificity of the assay. Samples were analyzed by real-time PCR according to the DNA results for each specimen. For example, if the sample was positive for HPV 16 DNA and negative for HPV 18 DNA, real-time PCR was done using only the HPV 16 primers.

A similar SYBR Green I real-time PCR technique, as described above, was used to identify the presence of the G3PDH gene to determine quality of the cDNA in the clinical samples used for quantification of HPV E7. For each amplification experiment, a final volume of 25 μl was used containing 2 μl of cDNA template, 12.5 μl of iQ SYBR Green Supermix (Bio-Rad), 1.25 μl containing 0.176 micromoles of G3PDH forward and reverse primers, and water. Primers used for G3PDH were 5'-acggatttggtcgtattggg-3' and 5'-tgattttggagggatctcgc-3'. They were designed to amplify a 230 bp fragment of G3PDH cDNA. The reaction was subjected to denaturation at 95°C for 2 minutes followed by 40 cycles of denaturation at 95°C for 30 seconds, annealing at 51°C for 30 seconds, and elongation at 72°C for 60 seconds. The presence of G3PDH in a sample indicated that we were able to get amplifiable product from that specimen; in this manner G3PDH was used as a quality control for each sample.

### Calculation of copy number from standard curve

The software with the PCR instrument calculates the standard curve for each run based on the C_T _for each standard (see Figure 2). Based on these values, a linear regression line is plotted and the resulting equation is used to calculate the log starting quantity and copy number for the unknown clinical samples.

## Competing interests

The author(s) declare that they have no competing interests.

## Authors' contributions

MES participated in the design of the study, carried out the experiments and statistical analyses, and drafted the manuscript. LMD participated in the design of the study and the performance of the experiments. ZC performed the design and production of the plasmids used in the study. MF participated in the design of the study. KAS conceived of the study, and participated in its design and coordination and helped to draft the manuscript. All authors read and approved the final manuscript.
